# Immature spinal cord neurons are dynamic regulators of adult nociceptive sensitivity

**DOI:** 10.1111/jcmm.12648

**Published:** 2015-07-30

**Authors:** Gabriel Rusanescu, Jianren Mao

**Affiliations:** MGH Center for Translational Pain Research, Department of Anesthesia, Critical Care and Pain Medicine, Massachusetts General Hospital, Harvard Medical SchoolBoston, MA, USA

**Keywords:** neuronal differentiation, chronic pain, neurotrophins, peripheral nerve injury, adult neurogenesis, nociception, BDNF, 7,8-dihydroxyflavone

## Abstract

Chronic pain is a debilitating condition with unknown mechanism. Nociceptive sensitivity may be regulated by genetic factors, some of which have been separately linked to neuronal progenitor cells and neuronal differentiation. This suggests that genetic factors that interfere with neuronal differentiation may contribute to a chronic increase in nociceptive sensitivity, by extending the immature, hyperexcitable stage of spinal cord neurons. Although adult rodent spinal cord neurogenesis was previously demonstrated, the fate of these progenitor cells is unknown. Here, we show that peripheral nerve injury in adult rats induces extensive spinal cord neurogenesis and a long-term increase in the number of spinal cord laminae I–II neurons ipsilateral to injury. The production and maturation of these new neurons correlates with the time course and modulation of nociceptive behaviour, and transiently mimics the cellular and behavioural conditions present in genetically modified animal models of chronic pain. This suggests that the number of immature neurons present at any time in the spinal cord dorsal horns contributes to the regulation of nociceptive sensitivity. The continuous turnover of these neurons, which can fluctuate between normal and injured states, is a dynamic regulator of nociceptive sensitivity. In support of this hypothesis, we find that promoters of neuronal differentiation inhibit, while promoters of neurogenesis increase long-term nociception. TrkB agonists, well-known promoters of nociception in the short-term, significantly inhibit long-term nociception by promoting the differentiation of newly produced immature neurons. These findings suggest that promoters of neuronal differentiation may be used to alleviate chronic pain.

## Introduction

Current chronic pain treatments have only limited effectiveness, largely because of an insufficient understanding of pain mechanisms. Recent reports have suggested that chronic pain may be linked to specific genetic defects, for example, in the case of fibromyalgia 1,2 or in c-kit 3, aldehyde dehydrogenase 2 (ALDH2) 4 and Notch3 5 transgenic mice, which would explain its extended duration. However, the large number of candidate genes and the absence of a clear mechanism make it difficult for this information to lead to effective treatments. Pain also occurs as a side effect of stem cell transplants used in spinal cord repair, which is thought to be the result of axonal sprouting 6,7, since the spinal cord environment promotes stem cell differentiation mainly into glia 8. Current chronic pain models, based on inflammation and synaptic plasticity, involve a significantly shorter time scale than that observed in most cases of intractable chronic pain, which can persist for years in the absence of any detectable injury.

The genes reported to regulate nociceptive sensitivity seem to be functionally unrelated; however, a closer look suggests that they may be all connected to neuronal differentiation. Fibromyalgia has been genetically linked to chromosome region 17p11.2-q11.2 2, which encodes some proteins also involved in neuropathic pain, such as Erk5 9, nitric oxide synthase 2 (NOS2) 10 and transient receptor potential vanilloid 2(TRPV2) 11. These proteins and many others encoded at this locus are also involved in neuronal differentiation, including Erk5 12, NOS2 13, TRPV2 14, kinase suppressor of Ras 1 (KSR1) 15 and Foxn4 16. ALDH2 17, c-kit 18 and Notch3 5, mentioned in relation to pain, are also expressed in neuronal progenitor cells and regulate neuronal differentiation. Any genetic variation that interferes with neuronal differentiation would result in incomplete neuronal maturation, including in the spinal cord dorsal horn layers that relay nociceptive information. These layers contain a significant number of inhibitory GABAergic interneurons and GABA is a major neurotransmitter in nociception, acting at both post- and pre-synaptic receptors 19. Immature neurons have increased intracellular Cl^−^ concentration and depolarize in response to GABA, in addition to showing increased excitability 20. Therefore, their connection to established nociceptive circuits would increase nociceptive sensitivity.

A similar process occurs during peripheral nerve injury, which has been associated with a change in the relative expression of the sodium-potassium-chloride cotransporter (NKCC1) and the potassium-chloride cotransporter (KCC2), resulting in increased intracellular Cl^−^ concentration and a depolarizing response to GABA 21,22. This suggests that some of the cells with altered NKCC1/KCC2 expression observed after nerve injury may be in fact immature neurons. This raises the possibility that chronic pain syndromes and enhanced pain sensitivity could arise from a neurogenic process, either developmental or induced by nerve injury, followed by incomplete neuronal differentiation, delayed or impaired because of genetic variations. Another chronic pain model suggests that peripheral nerve injury induces a loss of GABAergic inhibition in spinal cord laminae I–II, potentially as a result of neuronal death, resulting in a higher excitability of nociceptive pathways 23,24. However, this hypothesis was disputed as the number of neurons in lamina I was found unchanged for 2 weeks after nerve injury 25. Such discrepancy could potentially arise from the different time frames of these measurements 26. In addition, these two observations could be reconciled by the presence of a neurogenic process that continuously replaces dying neurons in the dorsal horn. The death of mature inhibitory neurons, combined with the integration of hyperexcitable immature neurons into sensory pathways would be overwhelmingly excitatory, resulting in a reduction in the nociceptive threshold. Genetic variations that interfere with the neuronal maturation process could explain chronic pain persistence long after recovery from acute nerve injury and remission of associated inflammation, as well as the large number of genes that appear to be involved in the regulation of nociception.

Here, we have tested the hypothesis that chronically increased nociceptive sensitivity is caused by immature spinal cord neurons. We have used a rat model of peripheral nerve injury, thought to mimic human chronic neuropathic pain, to demonstrate the presence of a transient wave of spinal cord neurogenesis, which suggests a dynamic temporal correlation between the production of immature neurons and nociceptive sensitivity. We also show that promoting neuronal differentiation with TrkB agonists concurrently reduces the expression of neural progenitor markers and nociceptive sensitivity.

## Materials and methods

### Reagents

Anti-fibroblast growth factor 2 (FGF2) neutralizing antibody, recombinant FGF2, recombinant epidermal growth factor (EGF; R&D Systems, Minneapolis, MN, USA), Erlotinib (ChemieTek LLC, Indianapolis, IN, USA), recombinant brain-derived neurotrophic factor (BDNF) (Prospec Bio, East Brunswick, NJ, USA), 7,8-dihydroxyflavone (TCI America, Philadelphia, PA, USA), 5-Ethynyl-2-deoxyuridine (EdU), azide-fluorescein (Jena Bioscience, Jena, Germany). Antibodies: Mash1, doublecortin (DCX), Notch3, nestin, calretinin, TrkB, glial fibrillary acidic protein, Skp2 (Santa Cruz Biotech, Dallas, TX, USA), NeuN (Millipore, Billerica, MA, USA), Cy3- and fluorescein isothiocyanate (FITC)-linked secondary antibodies (Jackson Immuno-Research, West Grove, PA, USA).

### Animals and surgery

Six-week old SD male rats were subjected to unilateral constriction injury of the right sciatic nerve (CCI) or to sham surgery, under anaesthesia with sodium pentobarbital. For direct spinal reagent administration, rats were first subjected to intrathecal catheterization and allowed to recover for 3 days. Rats with obvious neurological deficits after catheterization were excluded. EGF, FGF2, neutralizing FGF2 antibody, Erlotinib, BDNF or Vehicle (PBS) were administered every other day for the first 3 weeks and twice weekly thereafter. 7,8-dihydroxyflavone was injected i.p. with the same frequency. 1–3 animals per treatment were sacrificed at 0, 2, 4, and 6 weeks after CCI by perfusion-fixation for immunofluorescence analysis. The experimental design was randomized controlled laboratory experiment. Experimental protocols were approved by the IACUC Committee at Massachusetts General Hospital.

### Immunofluorescence

Rats were selected randomly from each treatment group for immunofluorescence analysis. After fixation and dissection, lumbar spinal cords were cryo-sectioned into transverse 35 μm-thick slices. Slices were permeabilized, incubated overnight with primary antibodies (4°C), washed in PBS and incubated 1.5 hrs in Cy3- and FITC-linked secondary antibodies. Slices were mounted on slides and imaged on fluorescence microscope equipped with FITC and Cy3 filters. Bleed-through was minimized by dual scanning on two different FITC-Cy3 filter sets with slightly different bandpass windows. Stereoscopic reconstruction and quantification were carried out using NIH ImageJ.

### EdU labelling

Six-week old rats, untreated or subjected to CCI for 1 or 3 weeks, were injected twice daily for 3 days with EdU (4 mg/kg i.p.). 0, 2 or 4 weeks post-CCI, the rats were killed by perfusion-fixation under anaesthesia. Spinal cord slices were permeabilized, incubated 15′ with azide-fluorescein (Jena Bioscience) and 1 mM Cu^+^. The slices were then washed and reprobed with primary antibodies followed by Cy3-linked secondary antibodies. The slices were mounted on slides using 4′,6 diamino-2-phenylindole (DAPI) mounting medium (Vectashield) and imaged by immunofluorescence.

### Behavioural tests

Nociception was assessed by the time course and intensity of allodynia and hyperalgesia. Rats were tested 1 day before CCI (Week 0) for allodynia and hyperalgesia, and individuals showing a larger than 15% differences from average were excluded. Rats were retested weekly for mechanical allodynia and heat hyperalgesia at the right hind paw subjected to CCI or Sham surgery. For allodynia measurements rats were placed in individual cages with wire mesh and accommodated for 30′. Ascending Von Frey filaments (0.007–300 g) were applied five times each to hind paws’ palmar surfaces. The mechanical threshold was defined as the lowest force resulting in at least three withdrawals. For hyperalgesia, a focused radiant light beam (Model 390; IITC Inc., Woodland Hills, CA, USA) was applied through a glass plate floor (Hargreaves test), at an intensity that resulted in a 12 sec. baseline withdrawal latency with 20 sec. cut-off, to the palmar surface four times at 10′ intervals. The average withdrawal time was defined as thermal withdrawal latency. For BDNF and flavone experiments, allodynia and hyperalgesia were tested daily during the first week and weekly thereafter. The experimenter was blinded to experimental conditions. Rats were placed in random order on the allodynia and hyperalgesia test surfaces by a person not involved in the experiment.

### Statistical analysis

A preliminary CCI experiment was run to determine sample size. A sample size of *n* = 8 rats per treatment was determined to be sufficient to pass the Shapiro–Wilk normality test and the equal variance tests. Three additional rats were included to be killed at various time-points during the experiment, for a total of 11 rats per treatment. Immunofluorescence was quantified using NIH Image J and averaged for five random slices per animal. Statistical significance was determined by two-tailed *t*-tests for *n* = 5 animals (each from a different experiment). For behavioural experiments, eight randomly selected rats per treatment were tested. Behavioural results were analysed using two-way repeated anova, considering two independent factors: treatment (CCI *versus* Sham, Notch3KO *versus* WT) and time (week=0–12), followed by the Holm–Sidak post-hoc test, for *n* = 8 animals. The reference group was assigned ‘Sham’ treatment at week 0 and the null hypothesis that there is no significant difference between the mean for each ‘treatment’ × ‘time’ pair and the reference group was tested. The significance of *F*-values for each anova analysis was tested against tabled *F*_critical_ values for alpha = 0.05. The initial CCI experiment, which tested the correlation of neurogenesis and nociception and did not involve intrathecal treatments, was performed five times. Experiments that involved intrathecal treatments were performed two times.

## Results

### Spinal nerve injury induces adult spinal cord neurogenesis

**Figure 1:**
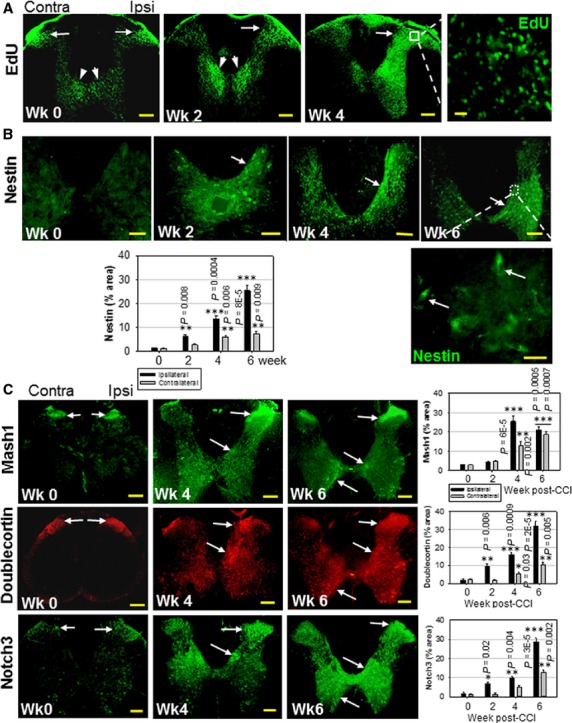
CCI Amplifies NPC Proliferation in the Adult Spinal Cord. (A) Time-dependent EdU incorporation (fluorescein, green) in the spinal cord, 0–4 weeks after unilateral CCI. Proliferating cells accumulate in the superficial spinal cord dorsal horn (arrows) and lamina X (arrowheads). (B) Time-dependent expression of nestin (FITC-green), 0–6 weeks after unilateral CCI in adult rats, showing the predominantly ipsilateral distribution of proliferating NPCs (arrows). Detail shows high density of nestin+ spindle-shaped cells (arrows). (C) Time-dependent spatial distribution of neurogenic markers Mash1, DCX and Notch3 after CCI in adult rats, 0–6 weeks post-CCI (arrows). Protein expression was quantified separately in the ipsilateral and contralateral halves of spinal cord grey matter. Data shown as mean ± SEM, *n* = 5 rats per condition. Statistical significance of changes in marker expression shown for ipsilateral and contralateral sides relative to sham-operated rats (two-tailed Student’s *t-*test). Protein expression levels in sham treated rats did not show any significant changes from control (week 0) and were omitted for simplification. Scale bars: 200 μm (A and C), 100 μm (B), 20 μm (A-detail), 10 μm (B-detail). Contra: contralateral; Ipsi: ipsilateral.

In the adult mammalian spinal cord, low level neurogenesis is present in the subependymal cell layer under normal conditions 27–30 or after injury 31,32 or disease 33,34. To test whether peripheral nerve injury increases spinal cord neurogenesis, we used a common experimental model of chronic neuropathic pain, the unilateral chronic constriction injury of the sciatic nerve (CCI) in adult rats 35. For the detection of proliferating cells, we used a chemical detection method based on labelling with thymidine analogue EdU 5,36. In control animals, basal EdU staining was concentrated in spinal cord laminae I–II and lamina X (Fig. 1A, week 0), suggesting that under normal conditions these areas undergo active cellular proliferation. This is consistent with a continuous ependymal layer gliosis (Fig. S1, day 0) 27 but also suggests the presence of a small pool of proliferating cells in laminae I–II 30. Within 4 weeks after CCI, the dorsal horn ipsilateral to nerve injury showed extensive cell proliferation (Fig. 1A). Some of the proliferating cells were likely reactive astrocytes (Fig. 1), as peripheral nerve injury has been typically associated with inflammation and astrocyte activation 37–39. Reactive astrocytes have stem cell properties and generate neural progenitor cells (NPCs) 40, and may also promote spinal cord neurogenesis by releasing growth factors 41,42.

The spinal expression of nestin, used previously to demonstrate stem cell proliferation in the adult spinal cord 30, was increased in rat spinal cord lamina X 2 weeks after CCI, reflecting NPC proliferation predominantly but not exclusively on the side ipsilateral to CCI (Fig. 1B). The time course and ipsilateral distribution of nestin expression correlated with EdU incorporation (compare Fig. 1A and B). The more intense EdU staining reflects cumulative EdU incorporation that includes proliferating NPCs and glia, while nestin expression is transient, as NPCs further differentiate in the upper dorsal horn layers. In subependymal layer cells, nestin coexpressed with F-box protein Skp2, a regulator of neural stem cell proliferation 43, suggesting that NPCs likely originate from the ependymal cell layer (Fig. S2A). Further from lamina X, nestin co-expressed with pro-neural transcription factor Mash1/Ascl1 (Fig. S2B).

**Figure 2:**
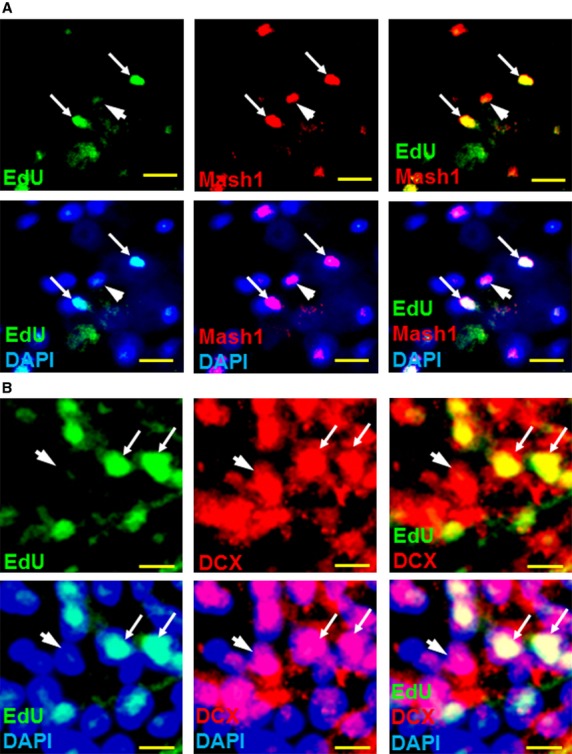
Proliferating spinal cord cells express neurogenic markers. (A) Immunofluorescence analysis showing the overlap of cell proliferation marker EdU (green, FITC) and proneural marker Mash1 (red, Cy3) in spinal cord dorsal horn, week 4 post-CCI, suggests that proliferating adult neural stem cells progress through neuronal differentiation stages similar to embryonic stem cells (arrows). DAPI fluorescence (blue) indicates all nuclei present. Some Mash1+ cells did not stain for EdU, suggesting they had stopped proliferating before EdU treatment (arrowhead). (B) Immunofluorescence analysis showing the overlap of EdU and neurogenesis marker DCX (red, arrows) demonstrate that newly generated NPCs progress through the final stages of neurogenesis within 8 days of EdU treatment. DCX+/EdU- cells (arrowheads) indicate NPCs that had already stopped proliferating before EdU treatment. Scale bars represent 20 μm (A), 10 μm (B).

Early and late stages of neurogenesis are reflected by the expression of Mash1 44,45 and DCX 46, respectively, as well as Notch3 5. Mash1 promotes DCX expression, thereby coupling neurogenesis with cell migration 47. Similar to EdU incorporation, Mash1, DCX and Notch3 were expressed in spinal cord laminae I–II in control rats (Fig. 1C, week 0), suggesting a continuous presence of partially differentiated NPCs in the superficial spinal cord dorsal horn layers. CCI further amplified Mash1, DCX and Notch3 expression preferentially in the dorsal horn ipsilateral to nerve injury. Corresponding phase contrast images are shown in Figure S3. Mash1 (Fig. 2A), DCX (Fig. 2B) and Notch3 5 expression partially overlaps with EdU staining, indicating the commitment to neuronal fate in some of the newly generated cells. By week 6 post-CCI, Mash1, DCX and Notch3 expression was significantly increased in the ipsilateral dorsal horns and in the ventral horns, suggesting that neurogenesis might also contribute to the motor abnormality (*e.g*. dystonia) associated with neuropathic pain.

### Injury-induced neurogenesis generates new neurons in the adult spinal cord

**Figure 3:**
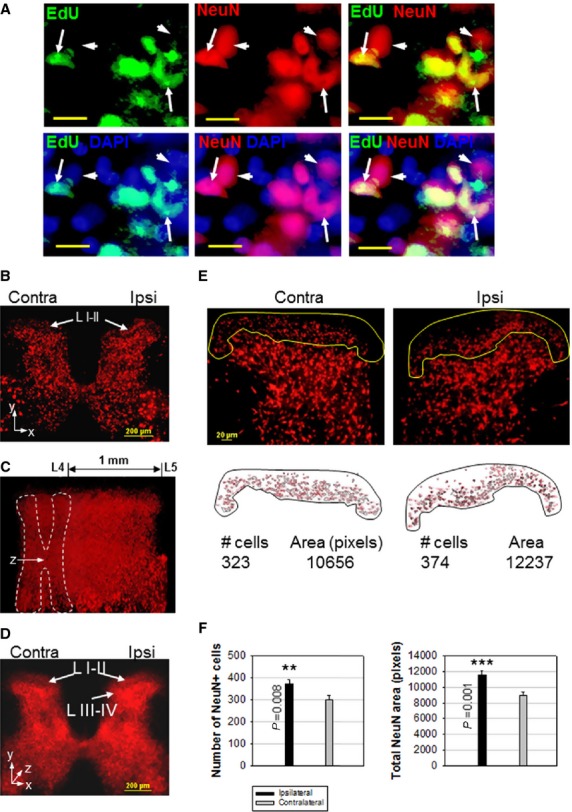
CCI Generates New Neurons in the Adult Spinal Cord. (A) Co-localization of proliferation marker EdU, neuronal marker NeuN and nuclear stain DAPI in the adult spinal cord (arrows), indicating newly generated neurons 4 weeks post-CCI. Arrowheads indicate cells stained by NeuN only, corresponding to pre-existing neurons. (B) Transversal slice of rat lumbar spinal cord, showing increased NeuN staining in ipsilateral laminae I–II (L I–II, arrows), 6 weeks post-CCI. (C) Stereoscopic reconstruction of a 1 mm-long spinal cord segment (region L4–L5) as a stack of 25 sequential spinal cord slices, rotated 60° around the *y*-axis (*z* = longitudinal axis), 6 weeks post-CCI. (D) Transversal view along z axis through the 25-section stack reveals areas with increased NeuN staining density in ipsilateral laminae I–IV (L I–IV) that correspond to areas of nociceptive transmission. (E) Individual slice quantification of ipsilateral/contralateral laminae I–II neurons (yellow perimeter), performed with NIH Image J. The number of NeuN-stained cells and total NeuN-stained areas are shown. (F) Quantification of CCI-induced changes in the number of NeuN-stained cells and in NeuN-stained areas in laminae I–II, 6 weeks post-CCI. Data shown as mean ± SEM, *n* = 5 rats. Statistically significant differences between ipsilateral *versus* contralateral laminae I–II (two-tailed *t*-test) are shown. Scale bars: 10 μm (A), 200 μm (B–D), 20 μm (E).

Increased NPC markers 4–6 weeks post-CCI suggested the possibility that some of the newly generated NPCs could become neurons and integrate into the nociceptive pathways. Some EdU-labelled cells in the dorsal horn had neuronal identity, as demonstrated by the partial co-localization of calretinin (CR) (Fig. S4), a marker for immature neurons 48, as well as NeuN, a mature neuron marker (Fig. 3A). This suggests that new neurons were generated in the spinal cord from proliferating progenitors during their 1 week EdU exposure. The co-expression of DCX and neuronal markers TrkB, NeuN or CR (Fig. S5) is further indication that endogenous NPCs can generate new neurons in the spinal cord. The post-CCI increase in ipsilateral TrkB expression 49,50 is consistent with the implication of neurotrophin signalling in nociception 51,52.

By week 6 post-CCI, spinal cord laminae I–II ipsilateral to the injured nerve showed a visible increase in the number of NeuN+ cells relative to the contralateral side (Fig. 3B). This increase correlated with the timing required for NPC proliferation, migration to the dorsal horn and differentiation into neurons. A tri-dimensional view of a 1 mm-long section of the lumbar spinal cord was stereoscopically reconstructed using a series of 25 sequential NeuN-stained slices (Fig. 3C). The xy projection of this 25-slice z-stack revealed that, in addition to laminae I–II, ipsilateral laminae III–IV also displayed increased NeuN staining, while the rest of the spinal cord remained largely unchanged (Fig. 3D). The lamina II/III boundary is clearly outlined anatomically, allowing the quantification of the number of neurons in laminae I + II (Fig. 3E). On average, ipsilateral laminae I+II had 21% more NeuN+ cells and a NeuN-stained area 27% larger relative to the contralateral side (Fig. 3F). The difference between the two measurements was likely due to overlapping cells. Thus, peripheral nerve injury produced a delayed increase in the number of NeuN+ cells within the ipsilateral spinal cord dorsal horn.

### Adult neurogenesis correlates with long-term changes in nociception

The long-term structural remodelling in the upper dorsal horn suggested the functional integration of newly generated neurons. The attenuation of GABAergic inhibition in the spinal cord dorsal horn associated with spinal cord injury and pain, assigned to changes in the relative expression of cation-chloride-cotransporters (CCC) 21,22, may result at least partially from an increased number of immature neurons, which would show a similar change in CCC expression 19. After maturation, many of these new neurons become inhibitory, which would then reverse the decrease in inhibition.

**Figure 4:**
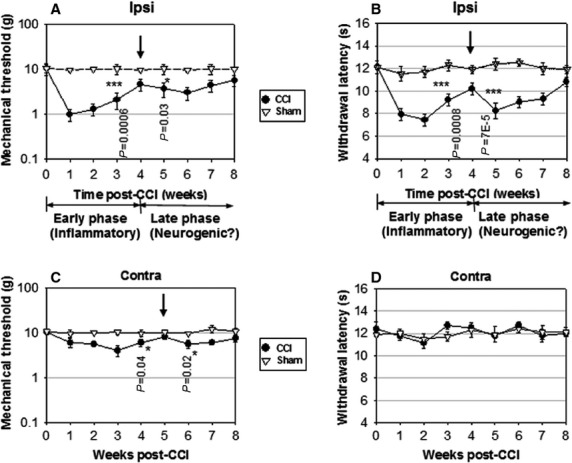
Temporal correlation between CCI-induced neurogenesis and late-phase nociception. The time courses of both mechanical allodynia (A) and heat hyperalgesia (B) on the ipsilateral side to CCI (Ipsi) show transient recoveries at week 4 (arrows). Nociception threshold levels at week 4 show statistically significant differences relative to weeks 3 and 5, indicating an inflection point suggestive of a change in mechanism. (C) Contralateral paw (Contra) shows a similar, albeit weaker, two-phase allodynia, with an inflection point at week 5 (arrow). (D) Contralateral hyperalgesia is negligible. Data shown as mean ± SEM, *n* = 8. Note: log scale representation may result in uneven depiction of ± error bars.

This hypothesis was tested by analysing the changes in behavioural response to nociception. On the ipsilateral side to CCI, the time course of CCI-induced allodynia (Fig. 4A) and hyperalgesia (Fig. 4B), two salient features of neuropathic pain 35, was characterized by an early-phase followed by a transient recovery around week 4 and a late rebound up to at least week 8 post-CCI (two-way repeated anova, CCI *versus* Sham, Fig. 4A: *F* = 64.52 > Fcritical = 5.32, *P* < 0.001, and Fig. 4B: *F* = 92.14 > Fcritical = 5.32, *P* < 0.001). The reactive gliosis that accompanied the early-phase had largely subsided to baseline by week 4 (Fig. S1), concurring with the transient recovery of allodynia and hyperalgesia. These results suggest that the neuroinflammatory response is unlikely to account for the prolonged time course of allodynia and hyperalgesia beyond week 4 post-CCI. The inflection point at week 4 post-CCI (Fig. 4A and B) suggests a change in mechanism between the early and late-phases of nociception. The early-phase correlates with glial activation, loss of GABAergic interneurons 26 and potentially with the differentiation of the local NPC pool in laminae I–II, while the timing of the late-phase correlates with the delayed increase in Mash1, DCX, Notch3 and NeuN expression. This two-phase pattern of neuropathic pain can be implicitly observed in previous reports 23,35,53, but became evident in this study when the signal/noise ratio was increased by displaying behavioural changes as a weekly variation on a semi-log scale. This hypothesis is supported by a similar variation in allodynia on the contralateral side (Fig. 4C, CCI *versus* Sham, *F* = 6.11 > Fcritical = 5.32, *P* = 0.029). The smaller increase in contralateral nociceptive sensitivity and the shift in intermediate recovery to week 5 are in agreement with the weaker expression of neurogenic markers in the contralateral dorsal horn. However, contralateral hyperalgesia was negligible (Fig. 4D, CCI *versus* Sham, *F* = 2.36 < Fcritical = 5.32, *P* = 0.11).

**Figure 5:**
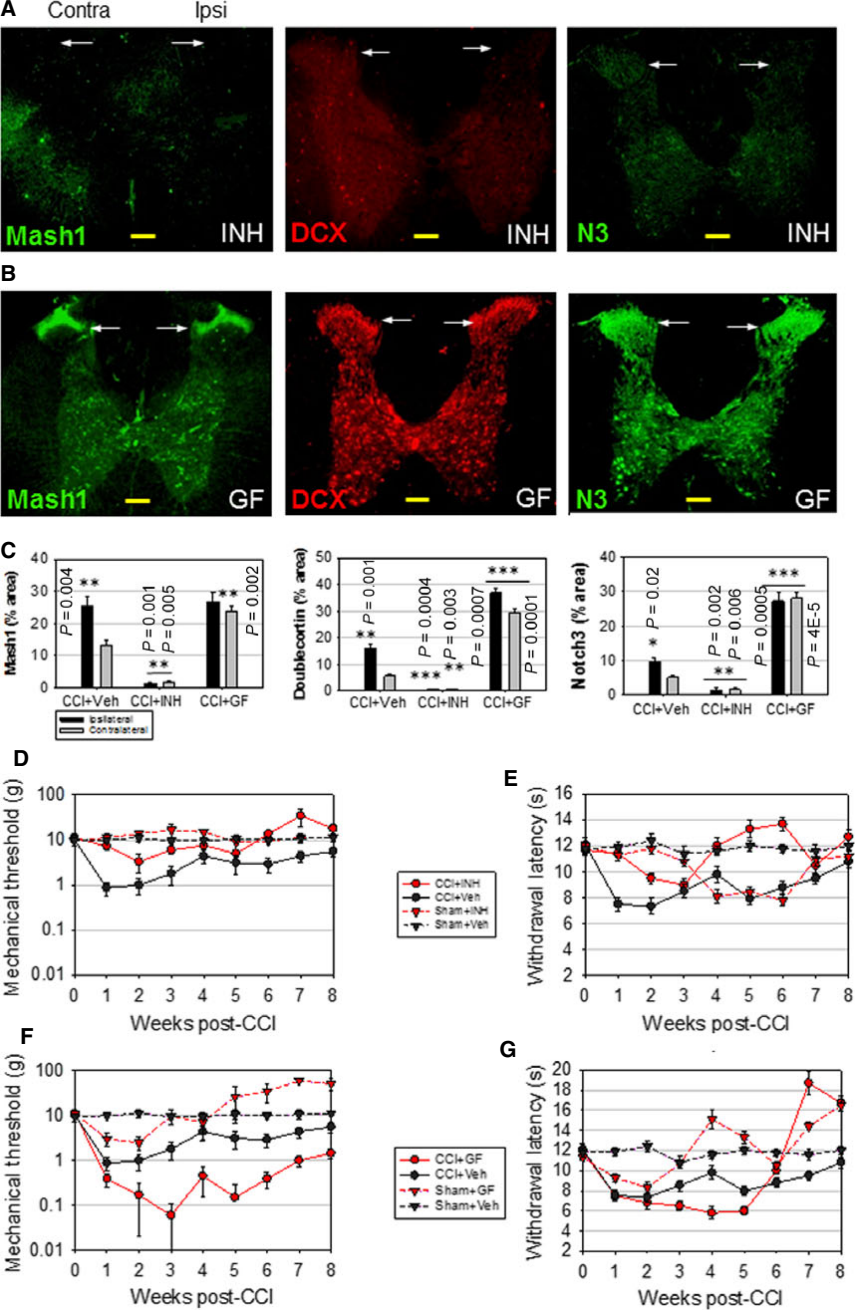
EGF/FGF2-dependent modulation of spinal cord neurogenesis corresponds to long-term changes in nociceptive sensitivity. (A) Immunofluorescence analysis of adult rat spinal cord, showing that inhibition of EGF/FGF2 signalling (INH) prevents the expression of neurogenesis markers Mash1, DCX and Notch3 (N3) in laminae I–II (arrows) 4 weeks post-CCI (compare to Fig. 1C). (B) FGF2+EGF treatment (GF) amplifies CCI-induced Mash1, DCX and Notch3 expression bilaterally, week 4 post-CCI. (C) Quantification of Mash1, DCX and Notch3 expression in the ipsilateral and contralateral halves of the spinal cord grey matter, in the presence of vehicle (Veh), INH or GF, week 4 post-CCI. Data shown as mean ± SEM, *n* = 5. Statistically significant differences shown for Veh_contra_ *versus* Veh_ipsi_, and between INH or GF treatments *versus* Veh, for corresponding ipsilateral and contralateral sides (two-tailed *t*-test). (D and E) Inhibition of neurogenesis correlates with decreased nociceptive sensitivity. INH eliminates late-phase allodynia (D) and hyperalgesia (E) relative to vehicle (CCI+INH *versus* CCI+Veh). (F and G) GF enhances mechanical allodynia (F) and heat hyperalgesia (G) relative to vehicle (CCI+GF *versus* CCI+Veh). Data shown as mean ± SEM, *n* = 8; scale bars: 200 μm.

The causal relationship between neurogenesis and long-term changes in nociception was tested through localized modulation of neurogenesis performed using its dependence on FGF2 and EGF 41,42. If the overall effect of immature dorsal horn neurons is excitatory, as suggested, the inhibition of neurogenesis would be expected to reduce nociceptive sensitivity. Indeed, the combined intrathecal administration of neutralizing anti-FGF2 antibody (10 μl, 0.2 mg/ml) plus EGFR inhibitor erlotinib (5 μl, 1 mg/ml) (INH) inhibits Mash1, DCX and Notch3 expression (Fig. 5A and C, compare to Fig. 1C, week 4), and concurrently prevents late-phase (week 5–8) allodynia (Fig. 5D, CCI+INH *versus* CCI+Veh, *n* = 8, *F* = 28.74 > Fcritical = 5.32, *P* < 0.001) and hyperalgesia (Fig. 5E, CCI+INH *versus* CCI+Veh, *F* = 100.4 > Fcritical = 5.32, *P* < 0.001). The partial effects of INH during the early-phase (week 1–4) are likely because of the inhibition of gliosis; however, gliosis is absent during the late-phase and cannot account for the late INH effects. The use of INH in Sham rats produced a delayed increase in heat hyperalgesia (Fig. 5E, Sham+INH *versus* Sham+Veh, *F* = 68.7 > Fcritical = 5.32, *P* <0.001), suggesting a net excitatory effect during the late-phase, potentially because of lower basal production of inhibitory neurons.

Conversely, intrathecal administration of FGF2 (10 μl, 0.1 μg/ml) plus EGF (5 μl, 0.1 μg/ml) (GF) accelerated and enhanced Mash1, DCX and Notch3 expression 4 weeks post-CCI on both ipsilateral and contralateral sides (Fig. 5B and C). These cellular changes were associated with more intense allodynia in CCI rats treated with GF (Fig. 5F, CCI+GF *versus* CCI+Veh, *n* = 8, *F* = 58.74 > Fcritical = 5.32, *P* < 0.001), while late-phase hyperalgesia shifted to earlier time-points, eliminating the transient recovery at week 4 (Fig. 5G, CCI+GF *versus* CCI+Veh, *n* = 8, *F* = 25.2 > Fcritical = 5.32, *P* < 0.001). GF treatment transiently decreased nociceptive thresholds in Sham rats, but the long-term effect was antinociception, suggesting an initial increase in neurogenesis, followed by NPCs maturation into an excess of inhibitory neurons (Sham+GF *versus* Sham+Veh, Fig. 5F, *n* = 8, *F* = 154.17 > Fcritical = 5.32, *P* = 0.011 and Fig. 5G, *F* = 10.47 > Fcritical = 5.32, *P* = 0.006). Since GF treatment did not extend CCI-induced gliosis relative to Veh (Fig. S6), the neuroinflammatory response cannot directly account for long-term behavioural changes. Instead, our data are more consistent with an extended excitatory activity by immature neurons produced by increased neurogenesis.

### Accelerating neuronal differentiation reduces nociceptive sensitivity

**Figure 6:**
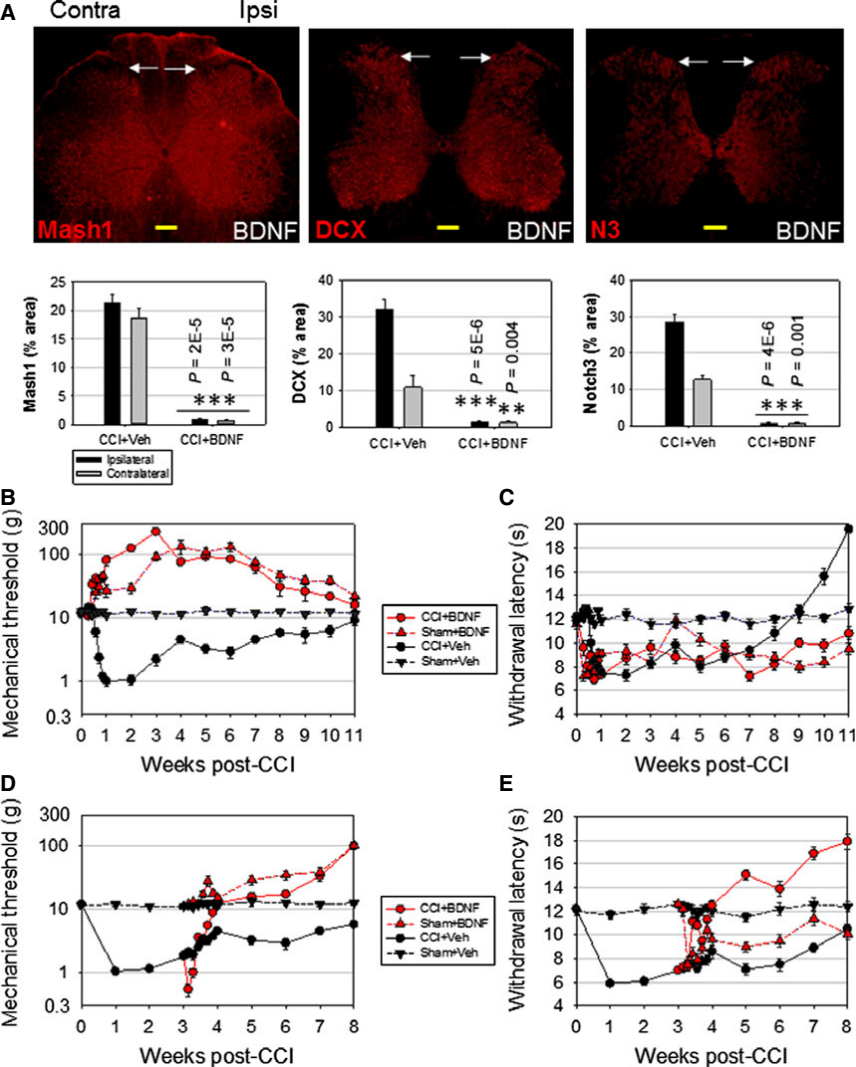
Adult spinal cord neurogenesis and nociception show similar BDNF dependence. (A) Immunofluorescence analysis of adult rat spinal cord, showing that BDNF treatment suppresses the expression of neurogenic markers Mash1, DCX and Notch3, week 6 post-CCI (compare with Fig. 1C, week 6). Quantification shown as mean ± SEM, *n* = 5 rats per treatment. Statistically significant differences shown for BDNF *versus* Veh, for ipsilateral and contralateral sides respectively (two-tailed *t*-test); scale bars: 200 μm. (B) BDNF treatment increases nociceptive threshold in both CCI and Sham rats. (C) BDNF administration extends CCI-induced hyperalgesia, preventing the typical recovery 2 months after CCI. (D) A delay in BDNF administration for 3 weeks post-CCI produces initially a brief decrease in nociceptive threshold, followed by a long-term increase. (E) A 3-week delay in BDNF administration increases long-term withdrawal latency in CCI rats, relative to administration beginning at day 0 (compare to C). BDNF-induced hyperalgesia in Sham rats remains similar to day 0 administration. Allodynia and hyperalgesia were measured daily during first week of BDNF administration, and weekly thereafter.

The increased TrkB expression associated with CCI (Fig. S5A) suggests it may play a role in adult-generated NPCs. Therefore, we tested whether TrkB activation accelerates the differentiation of newly generated NPCs, which would reduce the number of proliferating NPCs and the duration of the immature stage of new neurons. This would reduce long-term nociceptive sensitivity, in contrast with the short-term nociceptive effect of BDNF. Rats subjected to CCI and simultaneously treated with BDNF intrathecally (10 μl, 0.5 μg/ml every other day) showed reduced Mash1, DCX and Notch3 levels in lamina I–II (Fig. 6A, CCI+BDNF), in contrast to rats subjected to CCI only (compared to Fig. 1C, week 6). Corresponding phase contrast images are shown in Figure S7. Consistent with this decrease in immature neuron markers, CCI+BDNF rats showed a dramatic increase in nociceptive threshold (Fig. 6B, CCI+BDNF *versus* CCI+Veh, *n* = 8, *F* = 413 > Fcritical = 4.84, *P* < 0.001). A similar increase in nociceptive threshold was present in Sham+BDNF rats (Fig. 6B, Sham+BDNF *versus* Sham+Veh, *F* = 179.9 > Fcritical = 4.84, *P* < 0.001). The similar BDNF effects in Sham and CCI rats suggest that BDNF does not act through a CCI-related mechanism, such as preventing CCI-induced neuronal death, and that BDNF may be regulating nociceptive sensitivity by inhibiting neurogenesis, which occurs in both CCI and Sham rats. Contrary to its effect on allodynia, BDNF reduces the withdrawal latency to nociceptive heat stimulation in Sham rats (Fig. 6C, Sham+BDNF *versus* Sham+Veh, *n* = 8, *F* = 78.5 > Fcritical = 4.84, *P* < 0.001), similar to the effect of INH treatment (compare Fig. 6C and Fig. 5E). In CCI rats, BDNF prevents the recovery from hyperalgesia that normally begins in CCI+Veh rats around week 7 (Fig. 6C, CCI+BDNF *versus* CCI+Veh, weeks 7–11, *n* = 8, *F* = 124.3 > Fcritical = 6.61, *P* < 0.001).

Since BDNF treatment prevented NPC accumulation in the nociceptive layers, we investigated the outcome of delaying BDNF administration for 3 weeks post-CCI, to allow initial NPC proliferation and accumulation. This delay introduced a transient decrease in nociceptive threshold upon BDNF treatment, consistent with the synchronous differentiation of a large number of NPCs into highly excitable immature neurons (Fig. 6D, CCI+BDNF). This transient decrease was followed by a long-term inhibition of nociceptive sensitivity, consistent with neuronal maturation and an inhibitory response to GABA [Fig. 6D, CCI+BDNF *versus* CCI+Veh (weeks 3–8), *n* = 8, *F* = 318 > Fcritical = 6.61, *P* < 0.001]. In Sham+BDNF rats, the short-term increase in nociception was absent, reflecting the lack of immature neurons in the absence of CCI-induced neurogenesis. In the case of hyperalgesia, a 3-week delay in BDNF administrat-ion increased long-term withdrawal latency [Fig. 6E, CCI+BDNF *versus* CCI+Veh (weeks 3–8), *n* = 8, *F* = 349 > Fcritical = 6.61, *P* < 0.001], in contrast with the effect of simultaneous BDNF administration (compare Fig. 6E *versus* Fig. 6C, CCI+BDNF). BDNF action can be hindered by its inability to cross the blood-brain barrier, which requires intrathecal administration. Therefore, BDNF experiments were repeated by administration of a small molecule TrkB agonist, 7,8-dihydroxyflavone 54 (DHF, 1 ml, 70 mg/ml i.p. every other day; Fig. S8), which produced similar results. Coincidentally, the analgesic effect of flavones has been empirically known, however, they were thought to act through a mechanism overlapping with opiates 55–57.

## Discussion

### Increased nociceptive sensitivity corresponds to an increased number of spinal cord immature neurons

These results demonstrate that the late-phase of increased nociceptive sensitivity after peripheral nerve injury, which would correspond to chronic pain, correlates closely with the time course of spinal cord neurogenesis and subsequent neuronal differentiation and maturation in the spinal cord nociceptive layers. While the expression of neurogenic markers increases steadily in the ipsilateral half of grey matter over 6 weeks, their concentration in the upper dorsal horn layers shows a dramatic increase only beginning week 4. This delay likely represents the time required for NPC proliferation and migration to laminae I–II. The moderate increase in the contralateral expression of neurogenesis markers correlates with a weaker allodynia. CCI-induced neurogenesis is likely an amplification of a process normally present at lower intensity in rodent spinal cord 27–30. The newly generated neurons lead to extensive, long-term structural and functional changes in regions critically involved in nociceptive behaviour, which are an intrinsic indication of their survival and integration into existing neural networks. Otherwise, in the absence of synaptic contacts and neurotrophic support, newly produced neurons would undergo apoptosis relatively fast, in contradiction with the extended, 2–3 month long increase in the number of dorsal horn neurons observed. In fact, it is likely that many of the newly generated neurons do not form appropriate synaptic contacts and undergo apoptosis, similar to developmental neurogenesis. Experimentally, it would be difficult to distinguish these apoptotic neurons from mature neurons that die because of the peripheral nerve injury 26, or from the potential role played by autophagy 58. Our data suggest that spinal cord neurogenesis is likely similar to hippocampus 59,60 and olfactory bulb 61,62 adult neurogenesis. Even the pattern of reactive astroglia formed post-CCI on the medial edge of the dorsal horn, which coincides with the highest concentration of neurogenic markers, is evocative of the rostral migratory stream. The stochastic migration and connection of excitatory immature neurons to existing sensory and motor pathways is consistent with many features of chronic neuropathic pain including sensory and motor dysfunction as well as spontaneous pain beyond a typical dermatome distribution of the injured nerve. Accelerating NPC differentiation with TrkB agonists reduces the number of hyperexcitable immature neurons produced, decreasing nociceptive sensitivity. These findings support our hypothesis that genetic defects that interfere with neuronal maturation would increase the number of immature neurons in laminae I–II, increasing nociceptive sensitivity 5.

### Nociceptive sensitivity is regulated dynamically by continuous adjustment of spinal cord neurogenesis

The presence of proliferating cells under normal conditions in both lamina X and the superficial dorsal horn layers were indicated by EdU incorporation. This suggests that cells continuously generated in the subependymal layer migrate towards the dorsal horns 34,63 and gradually differentiate into NPCs that accumulate in the superficial dorsal horn layers. The presence of this pool of incompletely differentiated NPCs even under normal conditions suggests that neurons in these layers are gradually replaced during adult life. This continuous production of inhibitory interneurons throughout adult life might reflect a ‘nociception memory’ analogous to the olfactory memory 64, which could contribute to the continuous readjustment of the nociceptive threshold. Since the number of spinal cord neurons remains relatively constant throughout adult life, it is likely that the continuous spinal cord neurogenesis observed under normal conditions by other authors, and also shown here, reflects a continuous neuron turnover similar to the olfactory bulb.

CCI accelerates this process, amplifying both the apoptotic and the neurogenic processes. Previous reports have suggested that overall dorsal horn apoptosis after peripheral nerve injury can be as high as 20%, peaking 1 week after injury 26. This coincides with the timing of peak gliosis shown here, which would suggest an apoptosis-induced inflammation, as well as a phagocytic process. The phagocytosis of apoptotic neurons is a relatively fast process and may be another reason for the controversy on the loss of GABAergic neurons after CCI. Since the rates of these processes are unknown, a quantitative evaluation of post-CCI apoptosis is difficult. On the other hand, inflammation-induced gliosis could also promote neurogenesis 30, indicated by the spreading of neurogenic markers and EdU labelling. In addition, an unknown fraction of activated astrocytes can themselves play the role of NPCs 40. As a result of concurrent apoptosis, gliosis and neurogenesis, which occur with different kinetics, a numeric evaluation can only estimate the net neuronal variation in the ipsilateral dorsal horn. This CCI-induced wave of NPC proliferation and differentiation correlates temporally and functionally with the late-phase of neuropathic pain, in contrast to a robust but unsustained early-phase neuroinflammatory response. The induction of neural progenitor markers by CCI is not limited to the lumbar spinal cord region innervated by the sciatic nerve. We found a similar expression pattern is also present in the thoracic region, suggesting that NPC migration may also occur along the spinal cord longitudinal axis, possibly because of extended inflammation, consistent with secondary allodynia and hyperalgesia.

### The long-term reduction in nociceptive threshold corresponds mechanistically to the increased excitability of immature neurons

A ratio of the newly generated glutamatergic *versus* GABAergic neurons would be difficult to evaluate, since the markers commonly used for these neurons are somewhat ambiguous. For example, glutamine synthetase and GAD, often used to identify glutamatergic and GABAergic neurons, respectively, are also expressed in astrocytes 65,66. Mash1+ sensory neuron progenitors generate both glutamatergic and GABAergic neurons 67, both of which could undergo apoptosis in the absence of appropriate synaptic contacts. The overall effect of the integration of these neurons in nociceptive pathways would ultimately depend on their synaptic and network connectivity rather than on their numeric ratios. The emerging immature neurons initially have a high intracellular Cl^−^ concentration and depolarize in response to GABA. Therefore, their connection to existing nociceptive circuits would lower the nociceptive threshold. Certainly, some of these hyperexcitable neurons could be inhibitory or contribute to the excitation of inhibitory neurons. However, their effect would be limited by the desensitization of GABA receptors 68. In contrast, in some cases, glutamate receptors do not desensitize 69. As a result the net effect of the integration of immature neurons in nociceptive circuits would be an increase in excitability. As neurons mature, they become hyperpolarized in response to GABA because of decreasing intracellular Cl^−^ concentration, therefore, their integration into nociceptive pathways would over-compensate normal nociceptive sensitivity. This response reversal corresponds to the typical increase in nociceptive threshold observed 10–12 weeks after CCI. This neurogenesis-based model is supported by electrophysiological changes following spinal cord injury. After peripheral nerve injury 70 or spinal cord transection, a lamina I subpopulation of GABAergic neurons was identified that displayed more depolarized membrane potentials, larger spikes, steady-state outward currents and higher firing rates compared to control cells 71, properties which are also shared by immature neurons 72,73. Thus, the contribution of neurogenesis to the later phase of neuropathic pain may be regarded as a side effect of a normal repair mechanism that regenerates neurons lost because of spinal cord or nerve injury. This mechanism is compatible with, and may be mechanistically linked to short term, synaptic plasticity and inflammation-based pain models 11,38,39,74. Any genetic condition that interferes with this normal neuronal differentiation process would extend the immature excitable stage of neurons, possibly for the entire life of the individual. Such immature neurons may result from developmental defects or may be generated during normal or pathological spinal cord neurogenesis.

### Promoters of neuronal differentiation simultaneously reduce the number of immature spinal neurons and nociceptive sensitivity

The correlation between the number of immature neurons and long-term nociceptive sensitivity suggests that treatments that accelerate neuronal differentiation, such as BDNF, would reduce the duration and/or sensitivity to pain. Although neurotrophins are known promoters of nociception 51,52, BDNF-induced nociception only lasts for several hours 75. This is in agreement with our finding that delayed BDNF administration produces an initial surge in nociceptive sensitivity. However, this surge was absent when BDNF was administered simultaneously with CCI, likely because BDNF inhibits NPC proliferation from the beginning and does not allow a NPC buildup. This supports the idea that the transient surge in nociceptive sensitivity observed after delayed BDNF administration results from a synchronous NPC differentiation into a wave of immature neurons. Their subsequent differentiation into inhibitory GABAergic neurons would rapidly raise the nociceptive threshold, in agreement with our observation beginning day 5 after BDNF administration. Although some of these immature neurons may become glutamatergic neurons, previous reports have shown that BDNF selectively increases the expression of GABAergic markers in glutamatergic hippocampal neurons 76, and the number and length of dendrites and axon branches in GABAergic but not glutamatergic neurons 77. Therefore, a BDNF treatment as described here would selectively increase the number of GABAergic neurons and synapses, consistent with our behavioural observations. Previous electrophysiological studies seem to support this idea, showing that BDNF treatment of spinal cord dorsal horn increases the inhibitory drive to excitatory neurons 78. However, even simply accelerating neuronal differentiation, without changing the ratio of glutamatergic *versus* GABAergic neurons, can alone account for the rapid increase in nociceptive threshold shown here. Thus, BDNF treatment may shift the timing of the increase in nociceptive threshold typically observed 12–14 weeks post-CCI 35 to as early as 1 week post-CCI.

Different BDNF impact on allodynia *versus* hyperalgesia is consistent with previous observations 79. Allodynia appears to be influenced more by the hyperexcitability of new immature neurons, while hyperalgesia may be influenced more by the apoptotic loss of inhibitory neurons, both of which would result in chronically increased nociceptive sensitivity. This difference is eliminated when BDNF treatment is delayed post-CCI, initially allowing an unrestricted NPC proliferation. In that case, the large number of newly generated inhibitory neurons that mature after BDNF treatment reverses hyperalgesia as well.

## Conclusion

In conclusion, our results suggest that neuronal transition through immature stages normally associated with dynamic changes in excitability may regulate nociceptive sensitivity. Normal nociceptive levels may be maintained by a low steady-state neuron turnover. An increase in the number of hyperexcitable immature neurons produced as a result of injury, concentrated in the upper dorsal horns, would increase nociceptive sensitivity, followed by their synchronous maturation, which would reduce and even overcompensate the normal level of nociceptive sensitivity. Genetic variations that interfere with the normal neuronal maturation mechanism would extend the period of increased excitability. As a result, promoters of neuronal differentiation could be used as an alternative treatment for chronic pain. However, the effectiveness of such treatment would depend on the signalling hierarchy between BDNF and individual genetic variations that interfere with neuronal differentiation. Such structural plasticity-based mechanism would act in concert with inflammation and synaptic plasticity to regulate nociceptive sensitivity.

## Supporting Information

**Figure S1** CCI-induced astrocyte activation in the spinal cord is transient.

**Figure S2** Nestin co-localizes with other NPC markers in the spinal cord.

**Figure S3** CCI does not induce detectable macroscopic anatomical changes in the spinal cord.

**Figure S4** Proliferating spinal cord cells express neuronal markers.

**Figure S5** Neurogenesis marker DCX co-localizes with neuronal markers after CCI.

**Figure S6** GF treatment does not extend astrogliosis beyond week 4 post-CCI.

**Figure S7** BDNF treatment does not induce detectable anatomical changes in the spinal cord.

**Figure S8** TrkB agonist 7,8 dihydroxyflavone inhibits neurogenesis and nociceptive behaviour in a manner similar to BDNF.
