# Supplementary figures and images for: Immature spinal cord neurons are dynamic regulators of adult nociceptive sensitivity
Source: J Cell Mol Med. 2015 Jul 30;19(10):2352–64. doi: 10.1111/jcmm.12648 (PMC4594677; doi:10.1111/jcmm.12648)

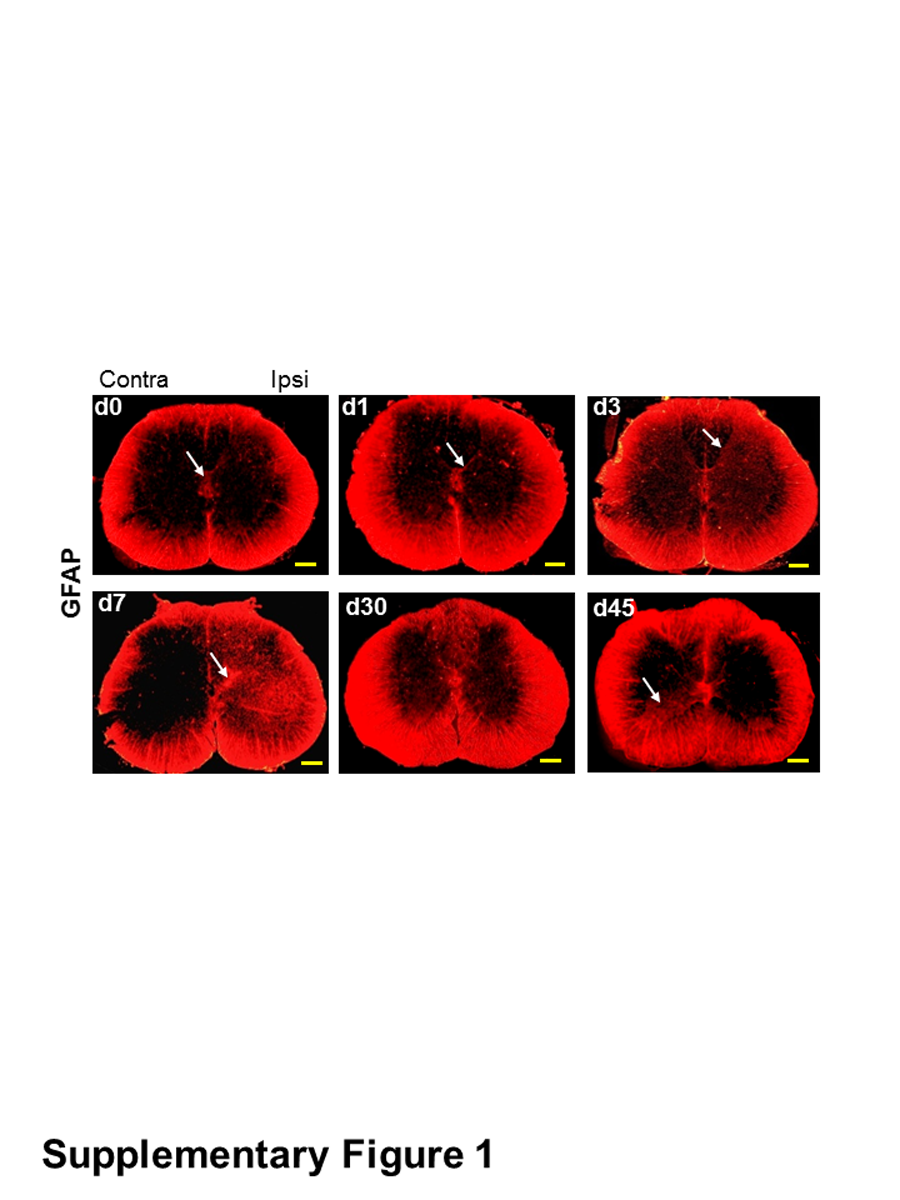

Supplement: Supplementary file 1 [file jcmm0019-2352-sd1.tif]

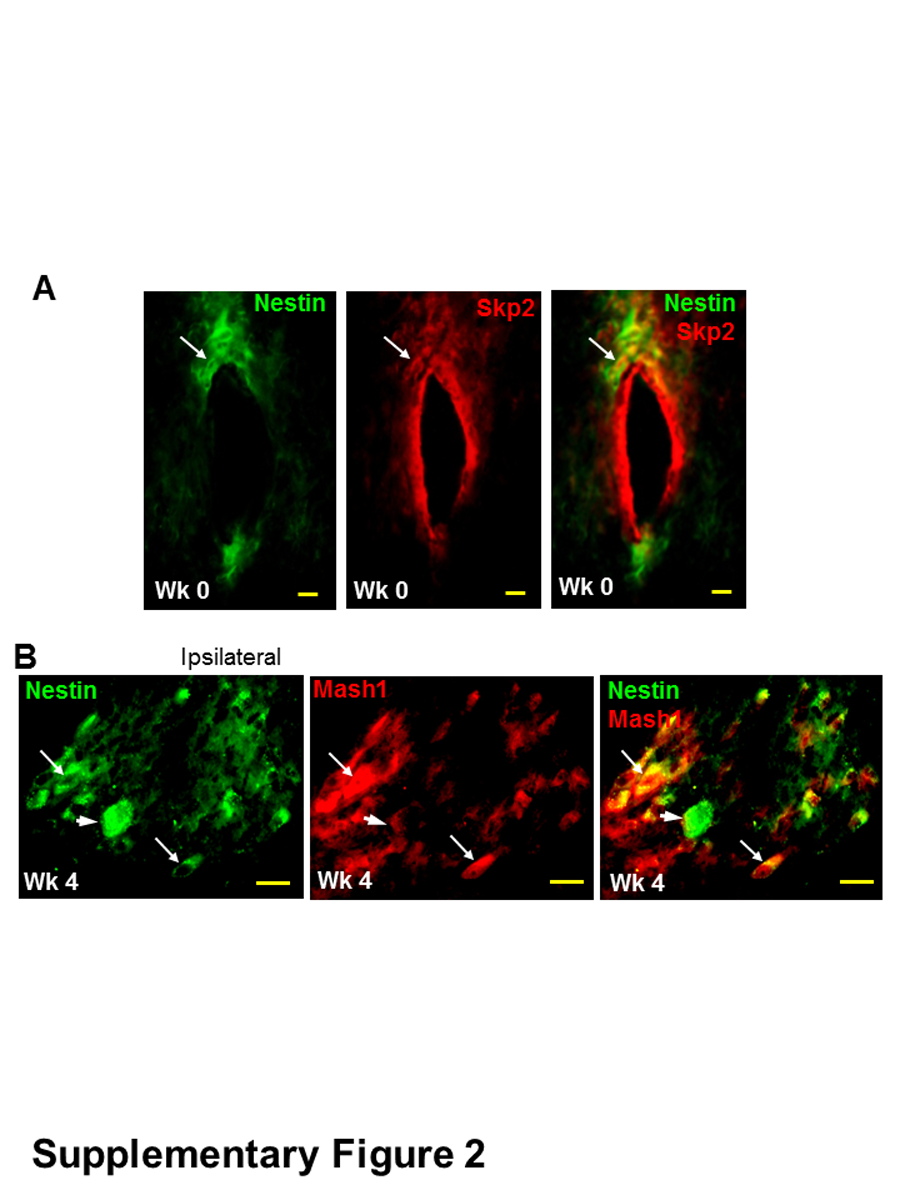

Supplement: Supplementary file 2 [file jcmm0019-2352-sd2.tif]

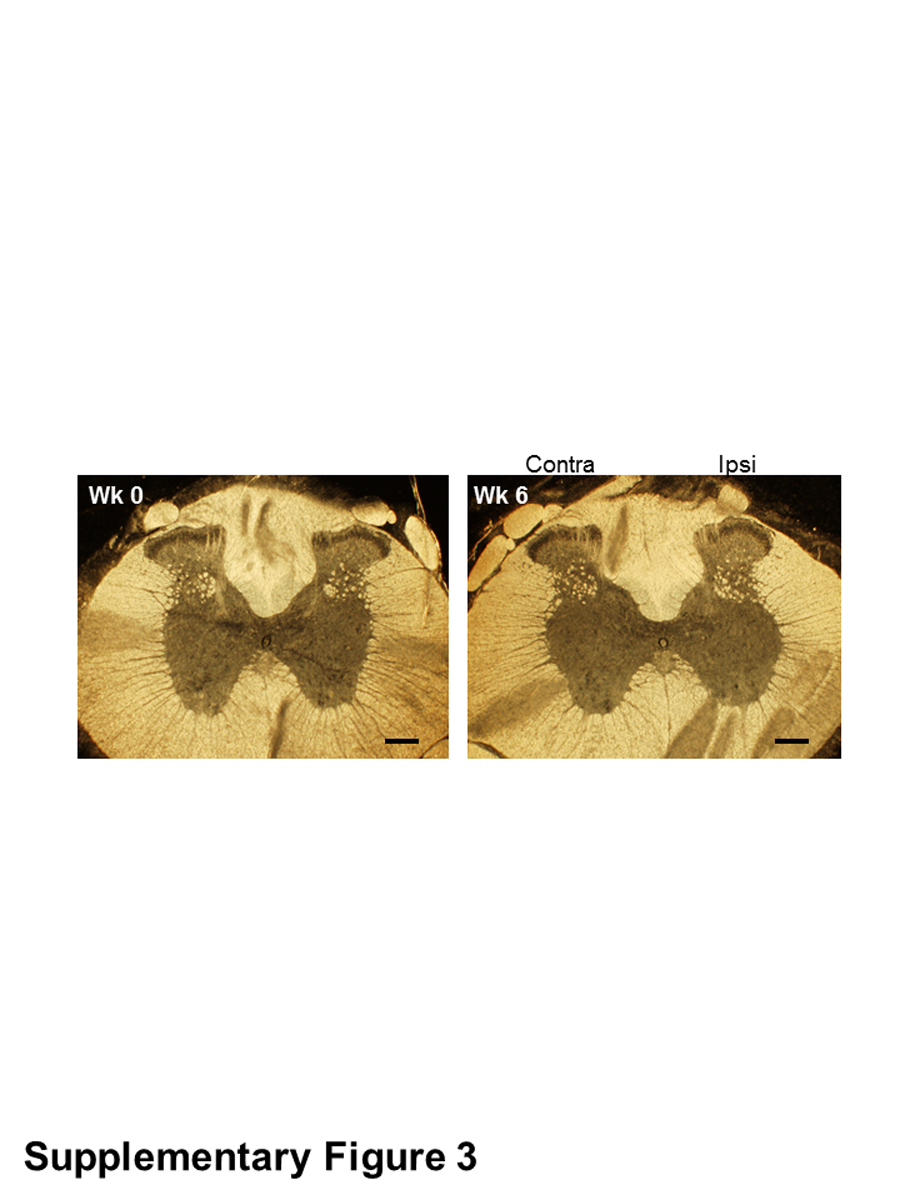

Supplement: Supplementary file 3 [file jcmm0019-2352-sd3.tif]

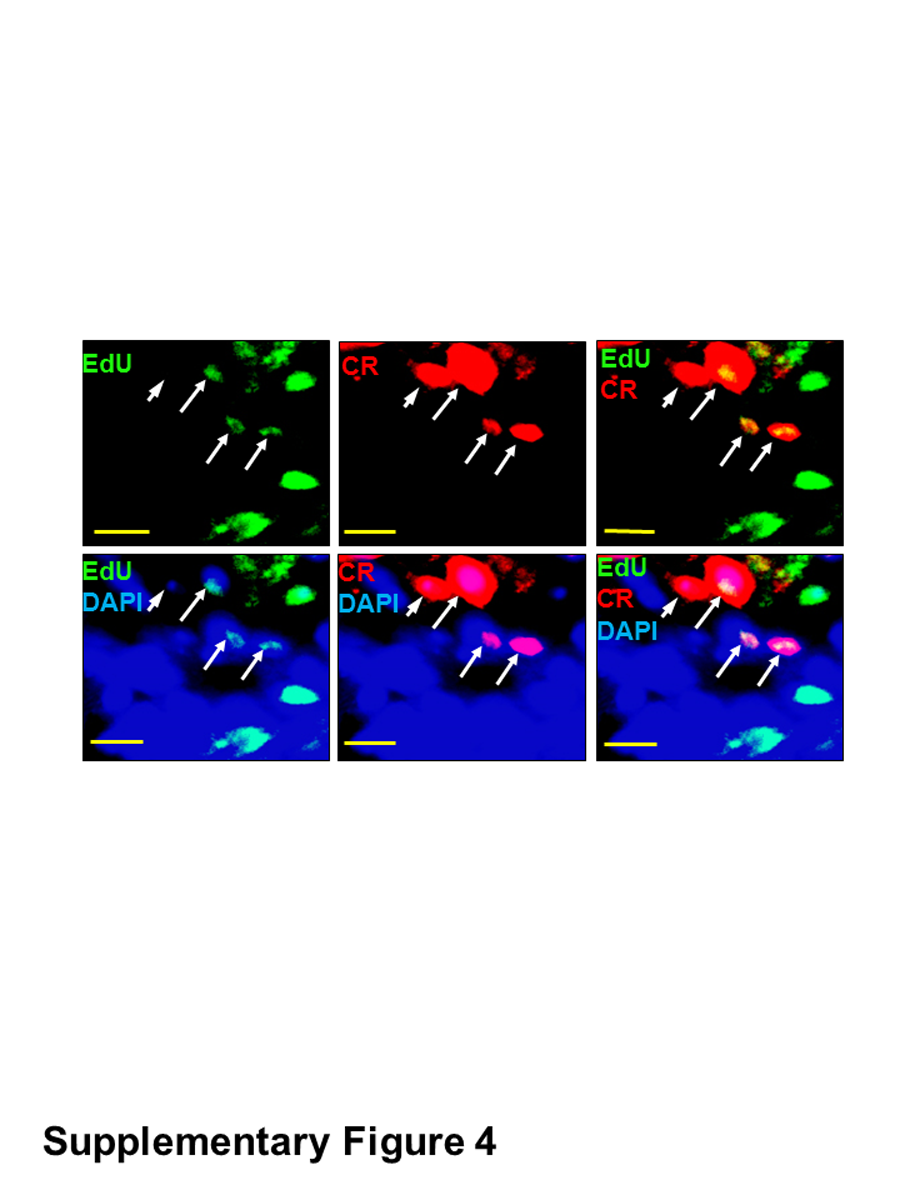

Supplement: Supplementary file 4 [file jcmm0019-2352-sd4.tif]

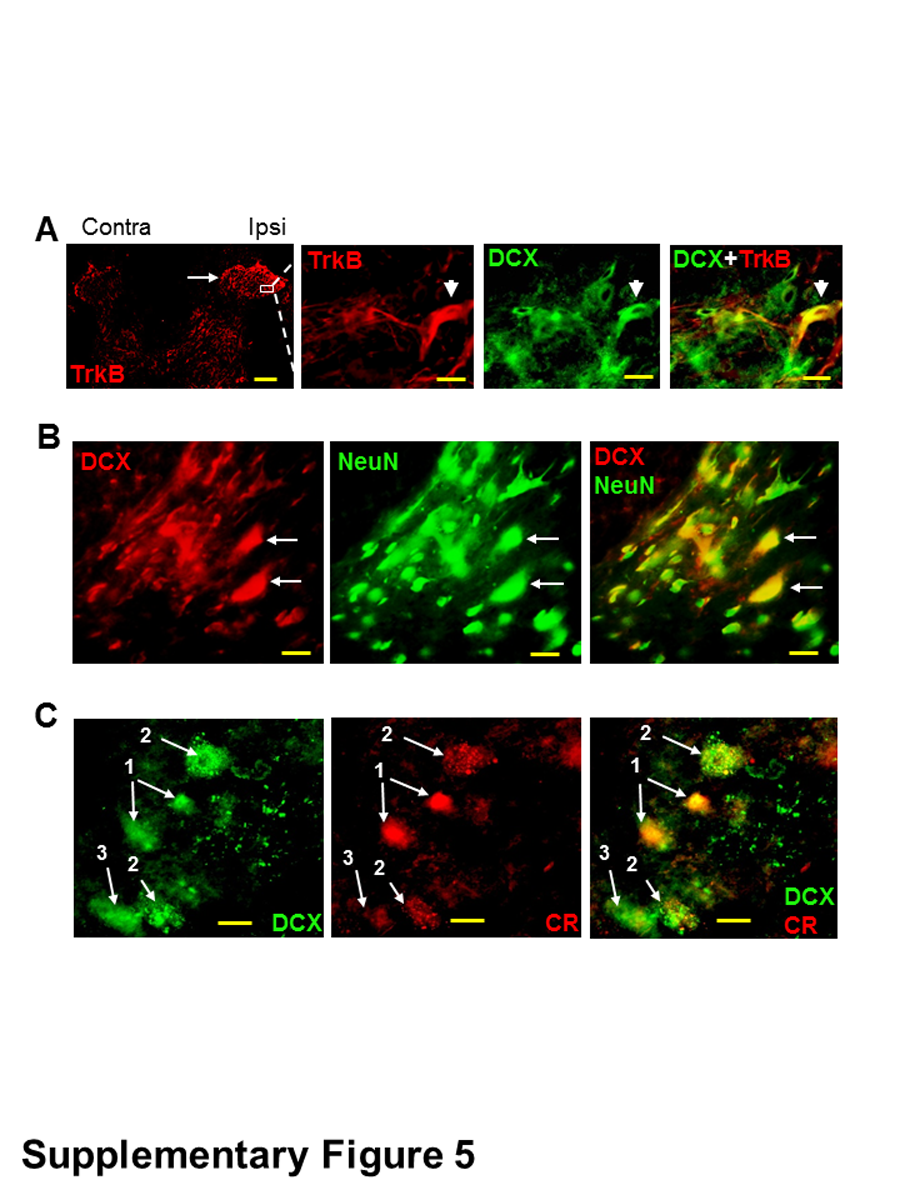

Supplement: Supplementary file 5 [file jcmm0019-2352-sd5.tif]

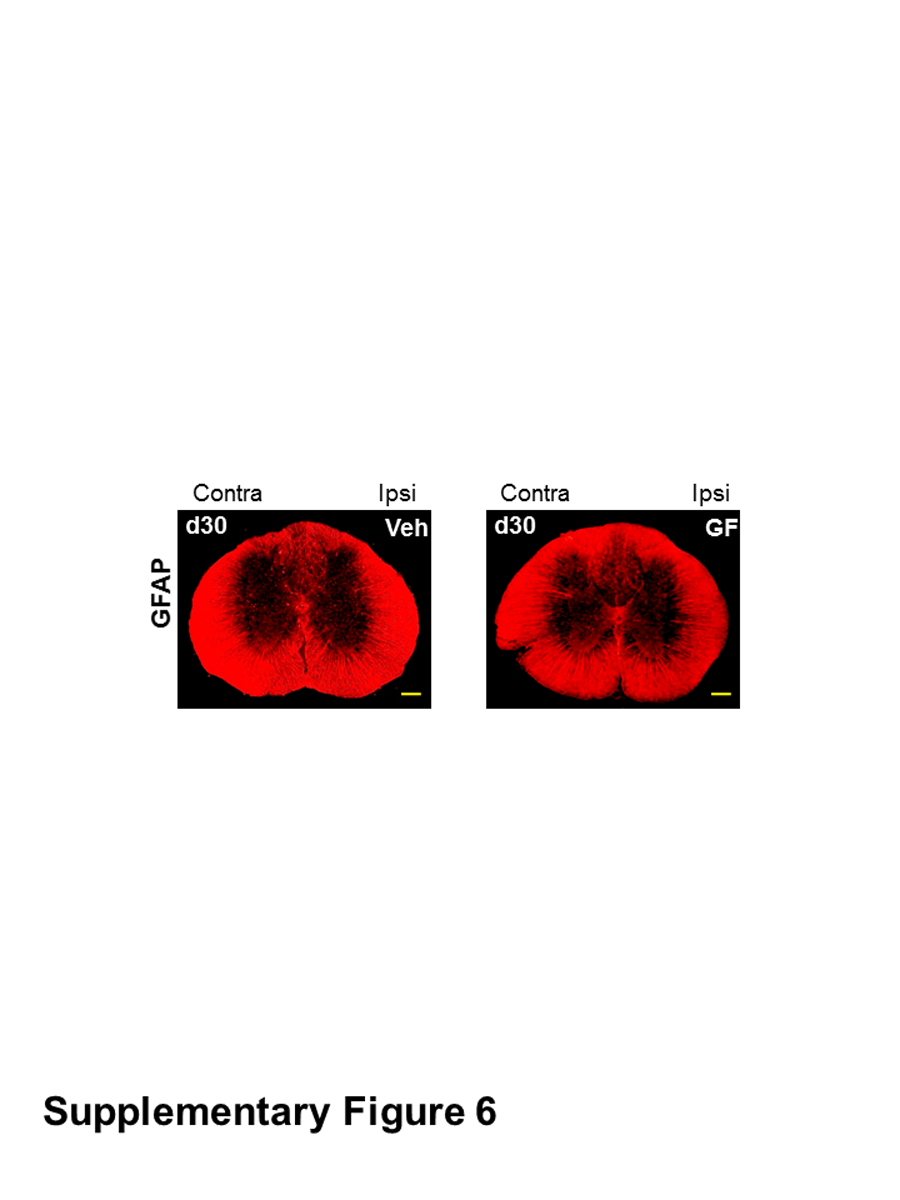

Supplement: Supplementary file 6 [file jcmm0019-2352-sd6.tif]

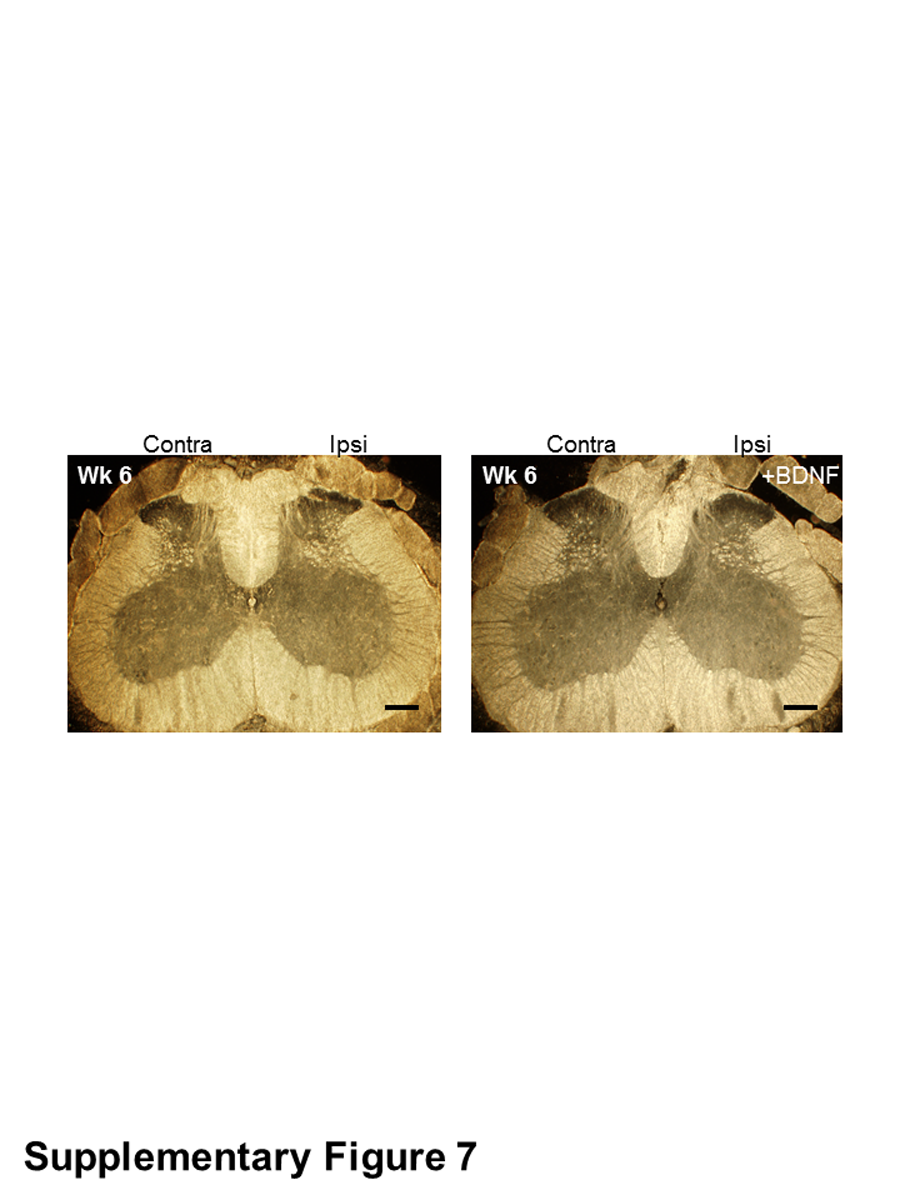

Supplement: Supplementary file 7 [file jcmm0019-2352-sd7.tif]

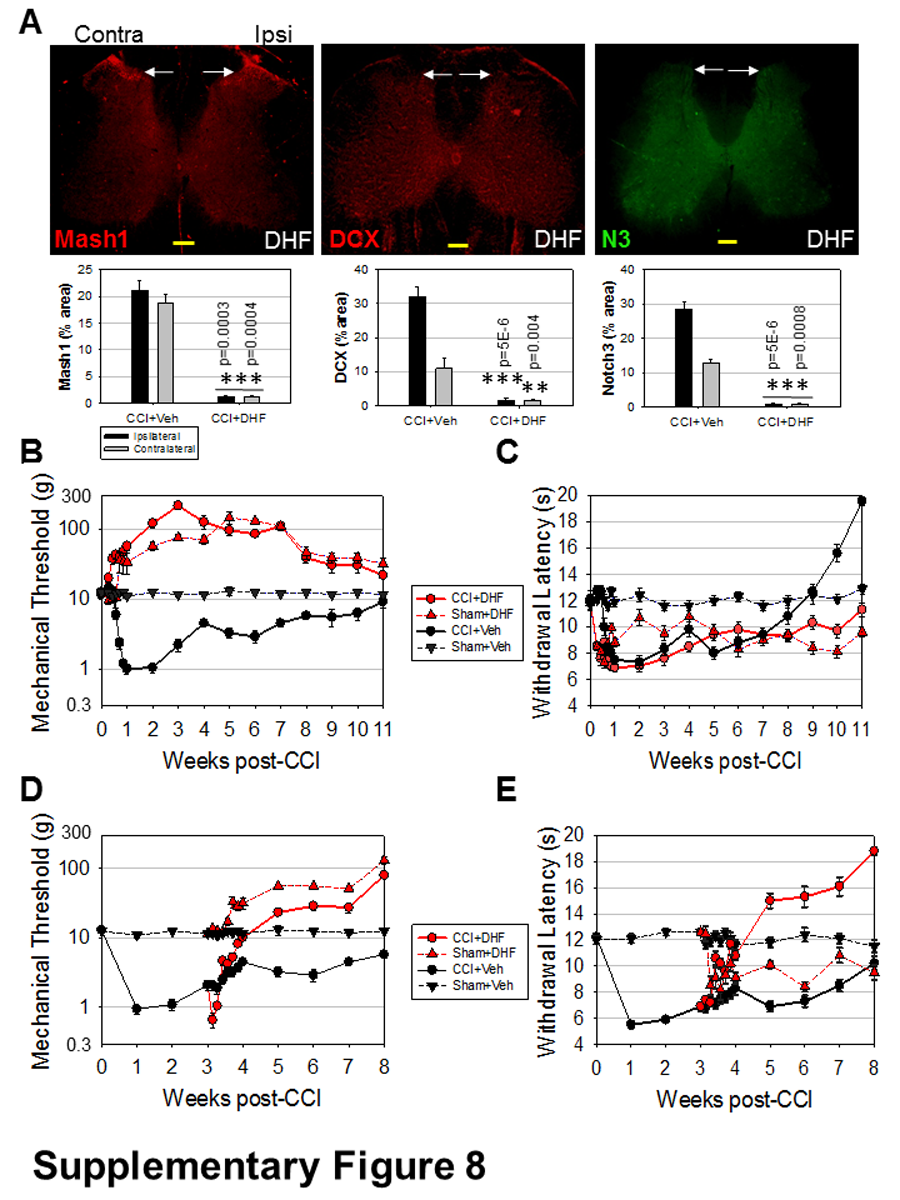

Supplement: Supplementary file 8 [file jcmm0019-2352-sd8.tif]
